# In silico metabolic profiling of non-*baumannii Acinetobacter* species uncovers conserved functions and provides first evidence of siderophore biosynthetic genes in *Acinetobacter junii*

**DOI:** 10.1038/s41598-026-60123-7

**Published:** 2026-06-30

**Authors:** Samuel O. Ajoseh, Ahmed F. Hikal, Hanka Brangsch, Abdul-Azeez A. Anjorin, Mohamed I. A. Hassan, Gamal Wareth, Kabiru O. Akinyemi

**Affiliations:** 1 Department of Microbiology, Faculty of Science, Lagos State University, P.M.B. 0001, Ojo, Lagos, Nigeria; 2 Division of Microbiology, National Center for Toxicological Research, U.S. Food and Drug Administration, Jefferson, AR USA; 3 Department of Bacteriology, Immunology and Mycology, Faculty of Veterinary Medicine, Benha University, Toukh, Egypt; 4 Institute of Bacterial Infections and Zoonoses, Friedrich‑Loeffler‑Institut, Jena, Germany; 5 Faculty of Medicine, Friedrich Schiller University, Jena University Hospital, Jena, Germany; 6 Institute of Infectious Diseases and Infection Control, Jena University Hospital, Jena, Germany

**Keywords:** Acinetoferrin, *Acinetobacter junii*, Non-baumannii *Acinetobacter*, Siderophores, *A. nosocomialis*, Metabolic pathways, Virulence, Antibiotic resistance, Computational biology and bioinformatics, Microbiology

## Abstract

The genus *Acinetobacter* is recognized for its metabolic versatility, which contributes to environmental persistence, virulence, and antimicrobial resistance. This study aimed to elucidate the metabolic pathways, particularly those associated with virulence and antimicrobial resistance (AMR), in non-*baumannii Acinetobacter* species. Genome-scale metabolic prediction of 19 *Acinetobacter* isolates identified 104 distinct pathways spanning 32 metabolic categories. Ninety-six pathways were conserved across all genomes, whereas a small subset exhibited species- or strain-specific distributions: four pathways occurred in 18 isolates, three were restricted to five *A. junii* isolates, and one was unique to 14 *A. nosocomialis* genomes. Amino acid metabolism was the most diverse (29 pathways), followed by lipid metabolism (14 pathways) and energy metabolism (8 pathways). The 96 conserved pathways supported essential cellular functions, including protein synthesis, energy production, and nucleotide metabolism. Notably, 20 pathways were associated with virulence, pathogenicity, and AMR, spanning lipid metabolism, siderophore biosynthesis, and nucleotide metabolism, among others. Eighteen of these pathways were conserved across all isolates and encompassed diverse metabolic functions, including protein N-glycosylation, heme biosynthesis, lipid IVA synthesis, fatty acid β-oxidation, tRNA maturation, triclosan resistance, and superoxide degradation. Interestingly, all *A. junii* isolates uniquely encoded siderophore biosynthesis systems. In silico analysis of the whole-genome sequences from the five *A. junii* isolates revealed the presence of genes sharing > 91% sequence identity with the acinetoferrin biosynthetic cluster (*acbABCD*) from *Acinetobacter haemolyticus.* Additionally, genes homologous to the acinetoferrin transport genes (*actB*, *actC*,* actA*, and *actD*) were identified. However, genes associated with the desferrioxamine E biosynthetic pathway were not detected in these isolates. The high degree of conservation of the acinetoferrin biosynthetic cluster in *A. haemolyticus* and *A. junii* suggests that *A. junii* likely produces acinetoferrin. These findings highlight the high degree of conservation of metabolic pathways in non-*baumannii Acinetobacter* species and represent the first report of the putative acinetoferrin biosynthetic gene cluster in *A. junii*.

**Supplementary Information:**

The online version contains supplementary material available at 10.1038/s41598-026-60123-7.

## Introduction

*Acinetobacter* is a diverse genus of Gram-negative, non-fermentative bacteria commonly found in soil, water, animals, and healthcare environments. It is recognized as an opportunistic pathogen in both clinical and non-clinical settings^1,2^. Despite this, non-*baumannii Acinetobacter* species have historically received relatively little attention, despite their presence across various ecological niches and their ability to cause infections in humans and animals^1,3^. Recent genomic studies show that several non-*baumannii Acinetobacter* species, including *A. junii*, are emerging One Health pathogens capable of transmission across clinical, animal, environmental, and aquatic sources^4–6^. This emergent threat highlights the need for a thorough characterization of the metabolic and physiological capacities of non-*baumannii Acinetobacter* species to better understand their adaptation, persistence, and virulence within host-environment systems^7^.

Metabolic flexibility plays a crucial role in *Acinetobacter*’s ability to survive in various ecological settings^7,8^. Surveys at the genus level have identified conserved pathways of central carbon metabolism, including the Entner-Doudoroff pathway, the pentose phosphate pathway, the tricarboxylic acid (TCA) cycle, the glyoxylate shunt, and gluconeogenesis. However, there is significant variability within and between species in how specific carbon sources, such as organic acids, amino acids, and complex lipids^7–9^, are used. This metabolic diversity reflects differences in genomic content and regulatory systems that help determine niche-specific fitness and survival strategies^7,9^. Our understanding of metabolic pathways and carbon source use in non-*baumannii Acinetobacter* species remains limited, mostly based on traditional phenotypic microarray studies or model organisms (*A. baylyi* ADP1 and *A. calcoaceticus*), rather than from comprehensive genome-scale systems-level analyses of isolates from clinical and environmental sources^7,10^.

In Lagos State, Nigeria, *Acinetobacter* species are involved in hospital-acquired infections, with reported prevalence rates of 1.92%−4.07% among hospitalized patients. However, detailed species identification and metabolic profiling of clinical and environmental isolates are still scarce^11,12^. Moreover, environmental and animal-derived *Acinetobacter* isolates, which represent the often-overlooked non-clinical parts of the One Health spectrum, remain largely uncharacterized at the genomic and metabolic levels, making it difficult to understand their epidemiology and transmission pathways^11,13^. Nonetheless, in silico metabolic pathway prediction, combining genome annotation and computational modeling offers a powerful way to explore microbial functional potential, especially in understudied taxa and regions^7,14^. Despite increasing interest in *Acinetobacter* ecology, data on the metabolic features of non-*baumannii Acinetobacter* species from sub-Saharan Africa are limited. Therefore, this study provides a predictive in silico analysis of metabolic pathways in non-*baumannii Acinetobacter* species, focusing on *A. nosocomialis* and *A. junii*, isolated from One Health sources in Lagos State, Nigeria. These analyses aim to improve our understanding of their ecological roles and potential public health implications.

## Materials and methods

### Ethical statement

Ethical approval for the use and collection of clinical and animal specimens was obtained from the Human Research and Ethics Committee of Lagos State University Teaching Hospital (LREC/06/10/1803) and the Lagos State Health Service Commission (LSHSC/2222/VOL III/68), with additional authorization for animal specimen collection granted by the Department of Veterinary Services, Ministry of Agriculture, Lagos State, Alausa (Health Research and Ethics Committee reference LREC/06/10/1803). Patient recruitment and all specimen collection commenced only after these approvals were secured. Human research was conducted in full accordance with the Declaration of Helsinki and its subsequent amendments with written informed consent obtained from each participant or, where relevant, from a legally authorized guardian. All animal procedures were conducted under the cited veterinary approval and in compliance with applicable animal welfare regulations.

### Sample sources and metadata of *Acinetobacter* species

In our previous study^15^, we sequenced 19 *Acinetobacter* isolates (five *A. junii* and 14 *A. nosocomialis* strains) from Nigeria (Table 1). The genome sequences were deposited in the European Nucleotide Archive (ENA) (BioProject PRJEB97386).

**Table 1:** Metadata of *A. junii* and *A. nosocomialis* isolates recovered from different sources in Lagos, Nigeria, used in this study.

FLI Code	Strain	Assembly accession	BioSample	Species	Host
24AR27661	KNS5	GCA_982397925.1	SAMEA121799044	*A. nosocomialis*	Cattle
24AR27662	KNS6	GCA_982397965.1	SAMEA121799045	*A. nosocomialis*	Cattle
24AR27663	OAN4A	GCA_982397935.1	SAMEA121799046	*A. nosocomialis*	Cattle
24AR27683	BGB056	GCA_982397905.1	SAMEA121799050	*A. nosocomialis*	Human
24AR27700	OAR8B	GCA_982397855.1	SAMEA121799051	*A. nosocomialis*	Cattle
24AR27704	OTABE01	GCA_982397835.1	SAMEA121799053	*A. nosocomialis*	Abattoir effluent
24AR27706	BGB026	GCA_982397845.1	SAMEA121799054	*A. nosocomialis*	Human
24AR27770	BGB038	GCA_982397885.1	SAMEA121799055	*A. nosocomialis*	Human
24AR27771	BGB047	GCA_982397875.1	SAMEA121799056	*A. nosocomialis*	Human
24AR27772	BGB154	GCA_982397955.1	SAMEA121799057	*A. nosocomialis*	Human
24AR27773	BGS066	GCA_982397945.1	SAMEA121799058	*A. nosocomialis*	Human
24AR27774	BGO079A	GCA_982397915.1	SAMEA121799059	*A. nosocomialis*	Human
24AR27892	BGB079B	GCA_982397985.1	SAMEA121799062	*A. nosocomialis*	Human
24AR27776	OAN6A	GCA_982397975.1	SAMEA121799060	*A. nosocomialis*	Cattle
24AR27668	OAN2B	GCA_982397895.1	SAMEA121799048	*A. junii*	Cattle
24AR27669	OAN3A	GCA_982397865.1	SAMEA121799049	*A. junii*	Cattle
24AR27701	CAE012	GCA_982397825.1	SAMEA121799052	*A. junii*	Abattoir effluent
24AR27791	LAU060C	GCA_982398005.1	SAMEA121799061	*A. junii*	Human
24AR27665	OAR6A	GCA_982397995.1	SAMEA121799047	*A. junii*	Cattle

### Bioinformatics analysis of sequenced non-*baumannii Acinetobacter* species

WGS and analysis of 19 non-*baumannii Acinetobacter* isolates were conducted as described by Ajoseh et al.^15^. Structural and functional annotation of the assembled genomes was performed using Bakta v1.10.3 with the v5.1 database^16^. Prediction of bacterial metabolic pathways was performed using *gapseq*^17^.

For validation of siderophore biosynthesis pathways, genomes and their corresponding annotations were screened for a selection of gene and protein sequences, respectively. The screening on gene level was conducted using ABRicate v1.0.1^18^, while DIAMOND v2.1.8 was used for protein screening of the genome annotations^19^.

## Results and discussion

**Fig. 1:**
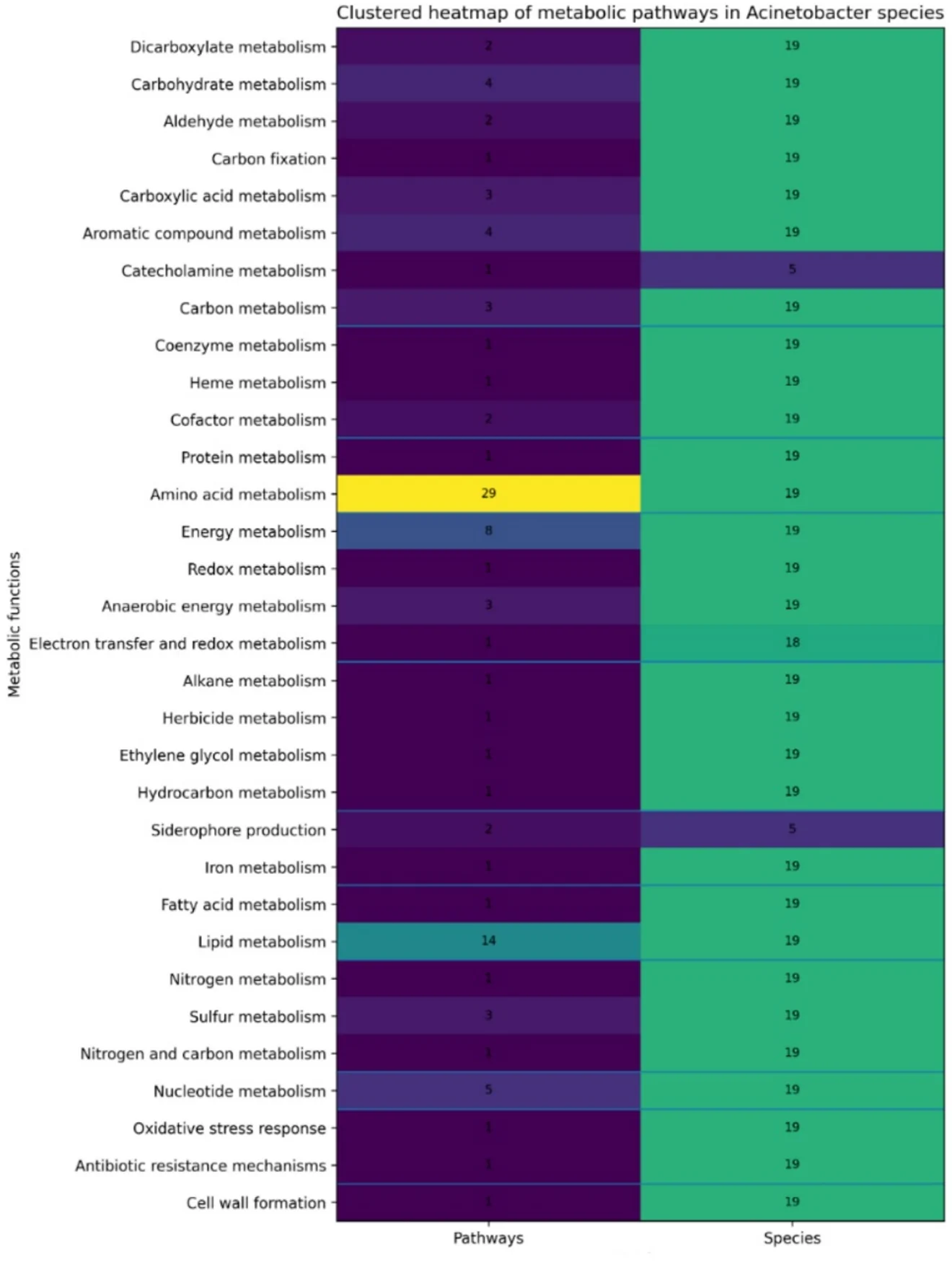
Heatmap illustrating the distribution of metabolic pathways across *Acinetobacter* isolates. Rows represent metabolic functions, while columns show the number of pathways identified and the number of *Acinetobacter*isolates containing each function. Color intensity follows a viridis gradient (purple to yellow), with increasing intensity indicating higher values.

Comparative metabolic profiling of 19 non-*baumannii Acinetobacter* species (14 *A. nosocomialis* and 5 *A. junii*) identified a diverse metabolic network comprising 104 pathways across 32 categories, with 96 pathways shared among all isolates (Fig. 1). These conserved pathways support essential cellular functions, such as protein synthesis, energy production, and nucleotide metabolism. Amino acid metabolism was the most diverse category (*n* = 29), followed by lipid metabolism (*n* = 14) and energy metabolism (*n* = 8). These findings are consistent with the known ecological versatility of *Acinetobacte*r in nutrient-limited niches and biofilm matrices, which, in turn, indirectly enhance transmission, stress resilience, and maintenance of antimicrobial resistance (AMR)^7,20–22^. Notably, 20 identified pathways were associated with virulence, pathogenicity, and antimicrobial resistance, spanning nine metabolic categories, with 18 conserved across all *Acinetobacter* strains (Table 2). These pathways included protein N-glycosylation for cell envelope assembly, heme b biosynthesis and bacterioferritin-mediated iron storage, which support respiration and homeostasis; lipid IVA synthesis and Kdo synthesis essential for LPS-dependent membrane integrity; and fatty acid β-oxidation and elongation, along with formaldehyde assimilation contributing to lipid maintenance. Additional conserved pathways included tRNA maturation metabolic pathways that maintain translational fidelity, and ethene biosynthesis III, which contributes to quorum-sensing-mediated biofilm regulation. Altogether, these findings reflect the importance of membrane integrity, iron homeostasis, and metabolic adaptability in facilitating host colonization and antibiotic resistance^33,34,38–40^.

**Table 2:** Metabolic pathways associated with virulence, stress tolerance, and antimicrobial resistance identified in non-*baumannii Acinetobacter* species using *gapseq*.

Metabolic category	Pathway/process	gapseq Pathway ID	No. of isolates	Biological function	Ref.
Lipid metabolism	Lipid IVA biosynthesis	NAGLIPASYN-PWY, PWY-8073, PWY-8245	19	Lipid precursor Synthesis: membrane integrity; virulence, and antibiotic resistance	^23^
	Kdo transfers to lipid IVA	PWY-8074, PWY-8246 (19), PWY2B4Q-6, PWY2DNV-1	19	LPS assembly virulence	^23,24^
	Fatty acid and formaldehyde metabolism	FAO-PWY, FASYN-ELONG-PWY, FORMASS-PWY	19	Energy production; and membrane synthesis	^25,26^
Iron metabolism	Ferroxidase	BACTERIOFERRITIN-PWY	19	Iron homeostasis oxidative stress n, and pathogenicity	^27^
Nucleotide metabolism	tRNA processing	PWYO-1479	19	Process tRNA maturation translational fidelity	^28^
	tRNA splicing	PWY-6689	19	Splice tRNA formation, protein synthesis, and pathogenicity	^28^
Antibiotic resistance	Triclosan resistance	PWY-7096	19	Protecting against triclosan and antimicrobial resistance	^29^
Hydrocarbon metabolism	Ethene biosynthesis III	PWY-6854	19	Quorum-sensing signaling	^30^
Oxidative stress response	Superoxide radical degradation	DETOX1-PWY	19	Protecting against reactive oxygen species and enhances antibiotic resistance	^7,31^
Protein metabolism	Protein N-glycosylation	PWY-7919	19	Protein folding, virulence, and immune evasion	^32^
Heme metabolism	Heme b biosynthesis	HEME-BIOSYNTHESIS-II	19	Electron transport, oxidative metabolism; and respiration	^33,34^
Siderophore metabolism	Desferrioxamine E biosynthesis	PWY-6375	5	High-affinity iron-acquistion	^35^
	Acinetoferrin biosynthesis	PWY-7989	5	Iron-chelating in iron-limited environments	^36,37^

Iron is a key cofactor in multiple biological processes in all living organisms. Iron homeostasis mechanisms, particularly heme b biosynthesis and bacterioferritin-mediated storage, are essential for *Acinetobacter* respiration under host-imposed iron limitation^33,34,41^. Mammals have evolved mechanisms to restrict the availability of free iron, thereby depriving invading pathogens of this essential metal^42^. To overcome iron scarcity, bacteria have evolved intricate strategies to acquire iron from their hosts. Many Gram-positive and Gram-negative bacteria produce siderophores, which have a high affinity for iron^43^. *Acinetobacter baumannii* harbors several siderophore biosynthetic pathways, but only acinetobactin has been shown to be essential for survival in iron-limited niches^44^. *A. haemolyticus* – a non-*baumannii Acinetobacter* – also synthesizes acinetoferrin in response to iron scarcity^45^.

**Fig. 2:**
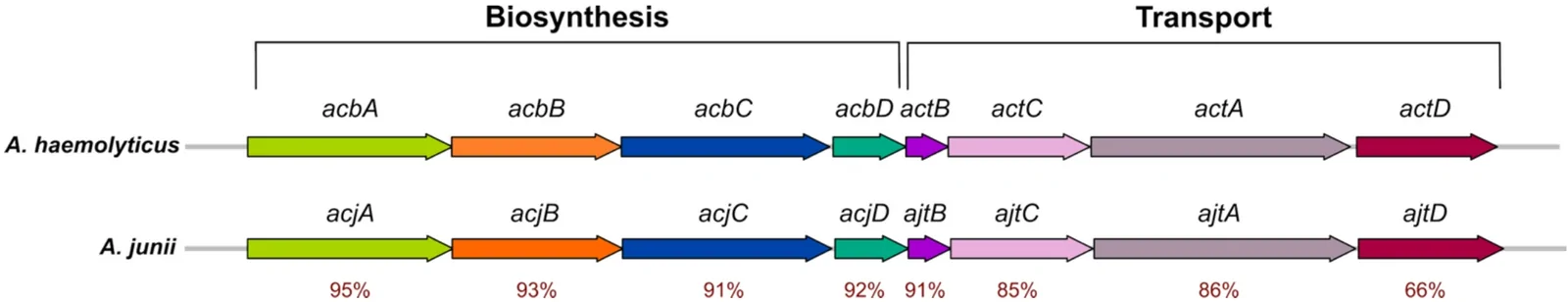
Schematic illustration of the conserved acinetoferrin biosynthetic and transport gene cluster. Homologs identified in *A. junii* compared to the reference genes from *A. haemolyticus* are shown for each locus. The *acbABCD* genes encode enzymes involved in acinetoferrin biosynthesis, while *actBCAD* encode transport proteins ^45^. Below, given in red, are the percentage identity on gene level between both species.

**Fig. 3:**
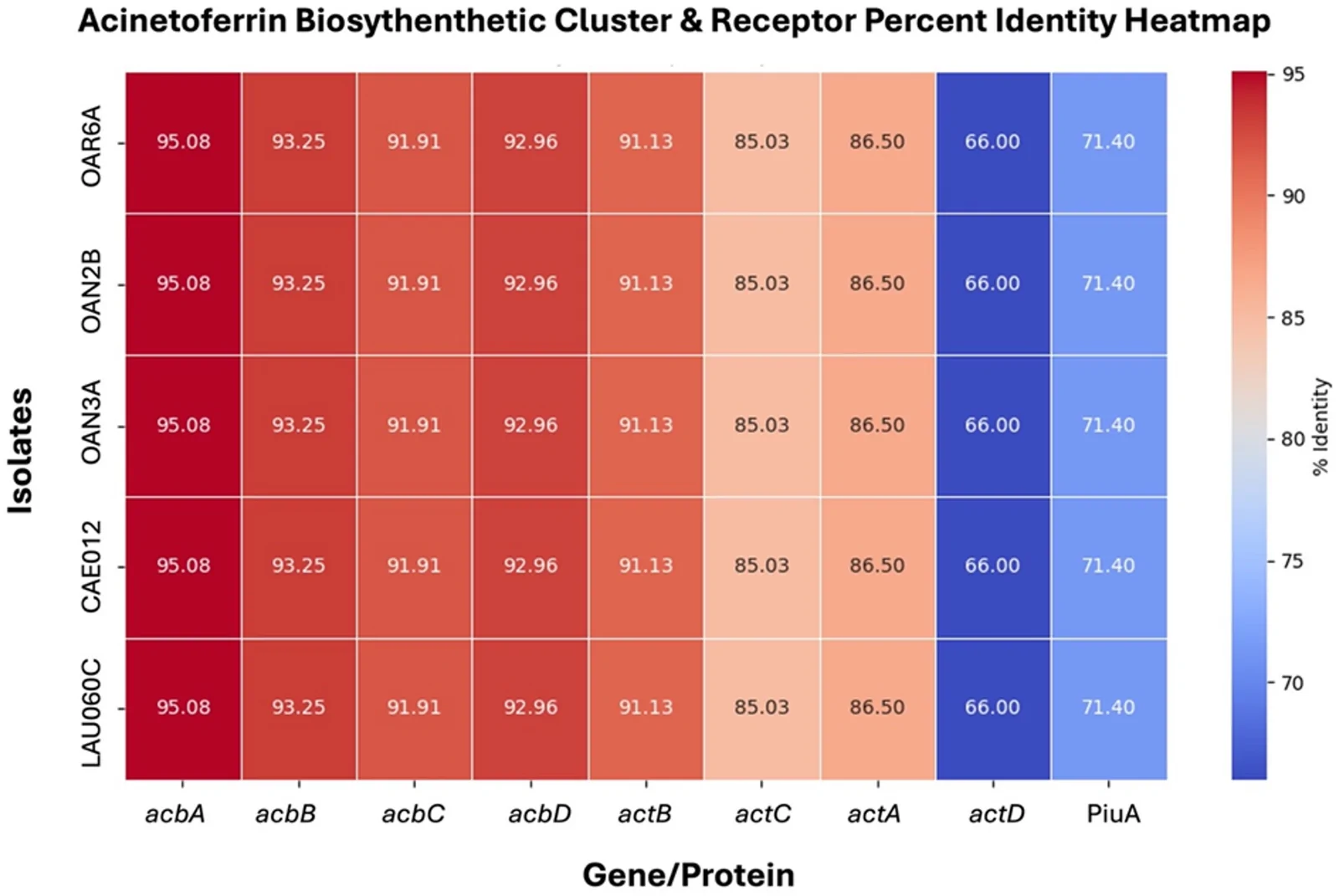
Heatmap illustrating the percentage identity of acinetoferrin biosynthesis genes (*acbA*,* acbB*,* acbC*,* acbD*), transport genes (*actB*,* actC*,* actA*,* actD*), and siderophore receptor protein (PiuA) homologs across five *A. junii* isolates. Rows represent the genes, while columns show each *A. junii* isolates harboring the genes. Color intensity follows a viridis gradient (red to blue), indicating increasing values.

Intriguingly, the screening with *gapseq* indicated that all five *A. junii*, but not *A. nosocomialis* isolates, encoded siderophore biosynthesis pathways for acinetoferrin (PWY-7989; 100% completeness) and desferrioxamine E (PWY-6375; 80% completeness), which are known from *A. haemolyticus* and *Streptomyces coelicolor*, respectively (Supplementary File 1)^45,46^. To confirm the presence of genes encoding these pathways in *A. junii*, we retrieved the nucleotide sequences of these genes and proteins from NCBI (Supplementary File 2) and performed an in silico screening for their presence across all *A. junii* isolates. We found that all *A. junii* isolates carried homologs of genes that encode acinetoferrin biosynthesis (*acbA*, *acbB*, *acbC*, and *acbD*)^45^, with sequence identities above 91% (Figs. 2 and 3). Additionally, these isolates harbored homologs of the four genes involved in acinetoferrin transport (*actB*, *actC*,* actA*, and *actD*), with identities of 91%, 85%, 86.5%, and 66%, respectively. We named the *acbABCD* homologs as *acjABCD* and the *actB*, *actC*,* actA*, and *actD* homologs as *ajtB*, *ajtC*,* ajtA*, and *ajtD*, respectively, where “*j*’ stands for “*junii*”. These data suggest that *A. junii* may possess the genetic potential to synthesize acinetoferrin, similar to *A. haemolyticus.*

Our in silico analysis also detected desferrioxamine E biosynthesis metabolic pathway (PWY-6375) across all five *A. junii* (Fig. 1). However, none of the genes encoding the desferrioxamine E biosynthesis pathway in *S. coelicolor* were found in the *A. junii* isolates. This discrepancy is likely because metabolic pathways prediction is generated by metabolic reconstruction (partial enzyme matches, promiscuous homologs, or pathway gap‑filling, which can introduce uncertain reactions) rather than confirmed, contiguous biosynthetic gene clusters^17^. Additionally, siderophore biosynthetic pathways are highly variable and often shaped by horizontal gene transfer, leading to patchy, incomplete, or noncanonical gene sets that still resemble known pathways at the functional level^47^. This makes false‑positive pathway predictions relatively common in siderophore‑rich taxa. Akhtar and colleagues detected the iron receptor proteins PiuA, PirA, CirA, and BauA in 112 *A. Junii* isolates^48.^ . To verify whether these receptors are conserved in our *A. junii* isolates, we conducted in silico screening for these proteins in the five *A. junii.* The data output identified hits for PiuA and PirA only, with shared identities of 71% and 29%, respectively (Fig. 3; data not shown for PirA**)**, suggesting a possible functional similarity of the PiuA homolog in *A. junii*. PiuA is a TonB-dependent siderophore receptor, which is involved in transporting siderophore-iron complexes across the outer membrane in *A. baumannii*^49^. Therefore, it is highly likely that *A. junii* uses AjtB, AjtC, AjtA, and AjtD along with the PiuA homolog to transport siderophores and their iron-bound forms across the inner and outer membranes. This finding aligns with the known importance of siderophores in virulence, environmental persistence, and competitive fitness^50–52^.

It is worth noting that the presence of the triclosan resistance (PWY-7096) and superoxide degradation (DETOX1-PWY) pathways in all investigated strains indicates adaptation to disinfectant exposure and host oxidative bursts, respectively, promoting environmental persistence and deep-tissue invasion^7,53^. Ethene biosynthesis (PWY-6854) may aid quorum sensing during biofilm formation^38^.

Collectively, this comparative profiling highlights ecological strategies that support *Acinetobacter* survival and pathogenicity. Additionally, our findings provide the first in silico evidence that *A. junii* carries genes for acinetoferrin biosynthesis. It should be noted that these findings exclusively rely on in silico predictions that require experimental validation. Further research is needed to determine whether *A. junii* produces a functional acinetoferrin. Also, future studies combining transcriptomic and metabolomic analyses under relevant stresses (hypoxia, iron limitation, antibiotic exposure) will be crucial for understanding the dynamics of these species-specific metabolic traits^54,55^.

## Conclusion

This study defines the metabolic architecture of *Acinetobacter* species as a complex network consisting of 32 metabolic categories and 104 pathways. A conserved core of 96 pathways was present across all 19 *Acinetobacter* isolates (14 *A. nosocomialis* and 5 *A. junii*), along with niche-specific adaptations in subsets of strains. More importantly, to the best of our knowledge, this is the first in silico report of putative genes encoding acinetoferrin biosynthesis in *A. junii*. These findings align with global studies on *Acinetobacter* metabolism while providing new insights into ecological and clinical specialization patterns. Overall, the results highlight the metabolic flexibility of the genus, which enables adaptation to diverse environments, thereby supporting persistence, virulence, and antimicrobial resistance. Consequently, these findings have significant implications for public health, as the metabolic adaptability and conserved pathogenic traits of these opportunistic bacteria challenge effective infection control and necessitate specialized monitoring and intervention strategies, particularly in clinical settings where multidrug-resistant *Acinetobacter* strains are becoming more common.

## Supplementary Information

Below is the link to the electronic supplementary material.

Supplementary Material 1

Supplementary Material 2

## Data Availability

The datasets analyzed during the current study are available in the European Nucleotide Archive (ENA) under the BioProject PRJEB97386. Further data are also available in the supplementary files.
