# Supplementary material for: In silico metabolic profiling of non-baumannii Acinetobacter species uncovers conserved functions and provides first evidence of siderophore biosynthetic genes in Acinetobacter junii
Source: Sci Rep. 2026 Jun 30;16:19911. doi: 10.1038/s41598-026-60123-7 (PMC13320182; doi:10.1038/s41598-026-60123-7)
Supplement: Supplementary file 2 — Supplementary Material 2 [file 41598_2026_60123_MOESM2_ESM.pdf]

I- Genes encode **acintoferrin** biosynthesis and transport:

- *acbA*
- *acbB*
- *acbC*
- *acbD*
- *actA*
- *actB*
- *actC*
- *actD*

DNA sequence

1- acbA

>AB661448.1:570-2354 Acinetobacter haemolyticus DNA, acinetoferrin biosynthesis gene cluster and transport genes, strain: ATCC 17906
ATGCAATCACTTGCTAATCGGTTGGCGTTACAACATTTCGTCAATGCATATACGCAAGAAACTGGCAAAG
GTCACTTGCTTCATAAAAGTCAGCAATCTCTTCAACTACAGACGTTTAGCCAAGGTTTGACGCTGTTATC
AATACCGATTACCTCTATTCAAGCACGTTGCTTTTTCCCTTTATCTTATGTGAGCTGTGTGGGGCGACAT
CGCTTAGCAGATCTTCCGCAAATTATTATCGATGAAAAAATTAAGGTATTTAGTCCTGTAGCAATTGTTG
GGTTGTTGCTAGAAGAACTTGTTCAAGAAGCCGCTGTTCATCTAGATGCTGCTTCATTAGTTGAAAAATG
GATTCAAAGTCGAGATGCGCTACAACAGTTTTTACAAAATCGTGAATATGAATTTGATGATTTAGTAAAA
GCAGGGCAAAGCTTTATTGAAACAGAGCAAGCTTTGATTTTAGGGCATAGTATGCATCCCGCGCCAAAAA
GTAGGACAGGCTTTGTTCATGAAGATTGGTTGAACTTTTCTCCTGAAAATAAAGGTAAGACTCAACTTCA
TTATTGGCTAGTGCACCAAGATTATATTGCAGAAGGTAGTGCGACTGATGAACCTATTTCAGCGCAATTA
AAAACCGCGTTACAGTGGTATTTATCTGAAAGTGATCTCAATTTATTAAAGACACATGCGGAATATAAGT
TACTTCCTTTACATCCATGGCAAGCGCGTTATTTACAGGGCAAAGCTTGGTTTGAACGATTAAAGCAAAC
AGGGCAACTGATTGATTTGGGCTTGCGTGGTTGGCAATTCTCTCCAACCACTTCCATACGCACATTAGCG
AGCTTCAATGCACCTTGGATGGTGAAGCCGTCACTTTCAGTGATGATTACCAATTCTATTCGAGTGAATT
TGGCCAAAGAATGTCATCGTGGTGAATTGACACATCGTCTGTGGCATAGTGAGTTTGGGCAAAATATTCT
TAAACAGTGTCCAAGTCTAAAAGCAGTCAATGATCCAGCTTGGATCGCTTTAAAAATCGATGATGAAGTG
ATCAATGAAAGTATTTGTATTTTCCGAGATCAACCATTCCATCCACAACAACAAGTCACTTGTATCGCAT
CTTTGTGCCAAGATCATCCGATTAAGCCTTTAAATCGTTTTAATGCCTTATTTGCAAAAATTGCGAAAGC
CCATCCTGATGCTGAAGTTTCAGAGATTGCTTCAGATTGGTTTAATCGGTTCCTCGAAGTCAGTTTGCAG
CCTTTGATGTATCTATACCATCGTTATGGTATGGCATTTGAGTCACATCAACAAAATGTATTATTAGAAC
TCGAAAACCATTTTCCTAAAACTTTATGGTTACGTGATAACCAAGGTTTTTATTATATTGAAGAATTTGC
CACTGAAATTCTGCAAGCACTTCCTGAACTGAAAGAAAAAGCCTTTGCGGTAGGTCCGAAAGCTTTTGTT
GATGAACGTTTTAGTTATTATTTCTTTGGCAATACTTTGTTCGGGATTATTAATGCCATCGGTGCAACAG
GCTATATTACTGAAGATGAATTGTTAGCGCATCTAACTGGATTTGTAGAAGAGCAATTACAGCAATATCC
TGAGAGTACCTTAATACAAGGACTACTGTTCAATTCAACGCTGCCTTATAAAGGCAACTTGCTGACACGT
TTGCATGAATTAGACGAGTTGATCGCACCTGTAGAAAATCAATCTGTTTACGTGCAACTGCCAAATCCGC
TGCGTATTTCACAGAAGGATGTCAGTTATGCTTGA

2- *acbB*

>AB661448.1:2347-3828 Acinetobacter haemolyticus DNA, acinetoferrin biosynthesis gene cluster and transport genes, strain: ATCC 17906
ATGCTTGATTTTATTGGAGTTGGTCTAGGGCCTTTTAATTTAAGTCTTGCTAGTTTGCTACAAAACAAAA
GCTCACTCAATTACGTGTTTTTTGAGCAAAAGGCGCAATTTGATTGGCATGCAGGTATGCAACTACCTAA
TACGGTATTACAAGTGCCATTTATGGCAGATTTGGTCTCGATGGTTGATCCAACCAGTCCATTTAGTTTT
TTGAATTATCTGCGTCATCAGCAACGTTTGTATAAGTTTTACTTTTTAGAGCAGCCACATATTCCACGTT
GTGAATACAACCATTATTGCCAATGGGTTGCAGAACAACTTGATTGTATCGAATACCAGTCGCAGGTTCT
AAAGATTGAACCTCAAACGATAGGTTTTAAAGTAGTCGTTGAATCAAACGGTGTTCAGCAAAGTTATTTA

TGCCGTCATTTGGTGGTTGGTAGTGGCAATGTGCCTTATTTGCCTGAATGTTTGGCAAAAATTCAGAAAA
TCCGTCCACAACAATGTCTTCATTCAGCACAGTATATGACGCATGCCGATACAGATTTACATGGCGATGT
GGTCGTACTTGGTTCTGGACAATCTGCAGCTGAAGTGTTTATCGACTTATTTGATGAACAGCAAGATACT
GTCAATCATCAGTTTGATTTGCATTGGTTTACGCGTTCTCAGGGTTTCTTCCCGATGGAGTATGCGCCAT
TAGGTTTGGAACATTTTAGTCCAGATTATGCTCAGCATTTTTATGATTTGACTGTGCAGCAAAAGGAACA
TCAGTTAAAGCAACAAGCCTTGTTATACAAGGGAATTAGCGCAAAAACCATTCGTGAAATTTATCAGAAA
TTTTATCACCGCAGTATTGCAGGTCAAAGCTTACAGACACATTTACATAGTCAATGTGATTTGAAAGATG
CAGAAGCATTGGATACACAAAAAATCCGACTGCATTTCCAGCATCGTGCTACAGCTCAGTCTTTTCATCT
CGACTGTGATTTTCTTGTAGCCGCGACGGGTTATTTCACGCCTGATTTTGGTTTTATGCAGTTGCTCAAG
CCATACATAGAGTTTGATCATAAACAGCGTTGGCAGATTACAGACGACTATCGTGTTGTACATCGGCTGA
ATGGTCATATTTTTGTTCAAAATCAGGAAATGCATAGTCATGGTGTCGGCACACCTGATTTAGGTCTTGG
TGCTTATCGTGCAGCAACGATCATTAATCAATTGCTTGCTGAGCCTCTTTATGAATTAGGCAATCAAGCG
CAGACATTCCAACACTTTAATCTCTCTCAAAATCCAAAAATTTGTATGGCAGATGACAAAGCACAATCAA
ACGAGTGCCTTTGCGAAAATTCTCCCAATAAAAACCATTCAATTTTCAAGAAAACAGAACAGAACGTGAT
GACGGCGCATCCTAATCGTCGTGGTGCAAACACTCGTACACAAACTGCTTCTGTACATTCAACATATGAG
AAACTGATATGA

3- *acbC*

>AB661448.1:3825-5642 Acinetobacter haemolyticus DNA, acinetoferrin biosynthesis gene cluster and transport genes, strain: ATCC 17906
ATGAACTTTGCAACTTTAAAAATTAATCAGCAGCAATGGCGTGCCGCAGGACAACGTCTTATTGAAATGG
CGATTGCAGAGTTTCTATACGAAGAAATTATTGAAGTTAAAGCATTGTCAGCAGGGCGTTATCGTTTAGA
TTTAGGCAATCGTCAGTATGAGTTTCAAGGAAATCAATATCTACTCGGACACTGGAATATTCAGGAAGCT
TCGGTACGCGATGTGACTCATAGTCAGGAAAATGATCAACCTGCTTGGAACTTACATGAGTTCATTGTCG
CAATGTCTGACAGTTCTAATGTTAAGCCATTTACCAAAGCGTATTTGATCAAAGAAATGAATAATACTTG
GCTTGCAGAAGCACATTTGTTCAATGAAACACGTTTGCCAAGTATAGCGGTTTTGACAGAGCCACATTAC
AAAGTTGAAGGCATGCTACGTGGTCATCCCTGGCTCATCATGAGTAAAGGGCGTATGGGTTTTGGTTATG
ATGATTATTTAAGCGCTGCACCAGAGCTTTCACCTGAAGTTAAAGTATTGTGGTTAGCCGTACATCGTGA
TTTGGCAGAGTATCGAAGTACTGAAGATTGGAGTGCCTGCGGTTTATATCAACATGAATTTGATGCCAAT
GAACTACAGCAATTCATCAATATTCTCCAAGAAAAAAATCTAGACCCGCAAAATTACTTTTTAATTCCAG
TACACGCATGGCAATGGCATCAATGGCTCGTTCCGACTTATGCCAATGAGATTGTCGATCAGAAAATCAT
TGAGCTGAATATCAGCCAAGATAGCTATGTGCCGATGCAATCAATCCGCACATTATGCAATACGTCAAAC
CTACAACGCCATTACATTAAGTTGCCTGTCAGTATTTTTAATACCGCTGTTTATCGTGGATTACCCTCAA
AGCGTAATCTAGCAGCTCCAGCAGTGACCGCATGGTTAAAAAGAATTCATCAACAAGATCAAGACTTACA
AAATACAGGTGTGATTTTCTTAGGTGAAGTTGCGACTTTAACTATCCATCAGCCTTGTTTCGATCGCATT
GATGGCGCACCGTATCAATTTAAAGAGCTGTTCGGTTGTTTATGGCGTGAAAGTGTTGATCGTTATGTTG
ATTCATCTCATCAAGTGTTATCTCAAGCAGCATTGTTACACCGTGATATTTCAGGTCAATCGATCTTGAG
CGTGTTGATCCAGGCATCAGGTTTAAGCCCTTTGGCTTGGTTAGCACAATTTGCTCAAGTAAGTATGAGT
CCATTATTACTTTGCTTATACCGCTATGGTTTGGCATTTTCACCACACGGTGAGAACACGATGTTGGTAC
ATGAAAATGGAGTACCAAAAGCTATGGTATTAAAAGATTTCATTGATGACATTAATTTGGTGGATGAGGA
TTTCCCTGAGTTAGCTCAGTTACCACCTGAAGCGGCTTTGTTATTACGACATGAGGCGACCGATCTAAGT
CATTTTATCTTCACTGGTCTGTTCATGGTTCATTACCGCTATATCTGTAATGTGTTCTTGCAAGACTATC
CTGAATATTCTGAGCTTGATTTCTGGCAGACCATTTCCAACACAATTGTTGAATTTAACCACAAACATCC
AGAACTGGCTGAACGTGCTGATAAATTCGCCATGCTGCGTCCTAACTACACCAAAATTTGCCTAAACCGT
GTACGTCTATTTACCACAAGTTATAACGATGAAGCAGAGCGTCCTGTGCCTGTCTTTCTTGATCCCATTG
CAAATCCAGTTAGCCCAGAAACATTGAAAACGTGGGCACAGCAACCTCGCCAAGCGAAAGTGGGCTAA

4- *acbD*

>AB661448.1:5670-6308 Acinetobacter haemolyticus DNA, acinetoferrin biosynthesis gene cluster and transport genes, strain: ATCC 17906
ATGAAAACAATCTCTCAGCAATTACCAGATTATTTTGAATACTTTGAAGATGGAACTCAGTACTACTTAC
GACAAGTGAAATATCCGCAGGATATTCCTTTATTGCACAAATGGATGCATGAACCACATGTGATTCCACA
GTGGCAGCTAAATAAATCGGAACTGGAGTTACAAGTTTACTTCGACAAAATGCTTGCAGATGACCATCAC

CGTTTATTGATCGTCGGAATTGATGGCAAAGATGTGGGCTATACCGAAATTTATGAGGGTAAACGAGATC
GTTTAGGCCGTTATTACGATGGTGATGACAATGATCTCGGTTGGCATTTGCTATTTGGTGACAAATCAGT
GTTCGGTAAAGGTTTTTTACGCCCAACTATTCGTTTACTCAGCTTCTATATTTTTGAGCACTCTAAAGCG
AAAAAAATTGTGGGTGAGCCAGATCATACCGTCAAACCTTATGCAGCAGTGGTGGCAGAGCTTTGTTATG
AAAGCCAACGTTTAATACCGATGCCTGAAAAAACAGCGATGTTGTACTACTGCTTTAGAGAAACTTTTTA
TCACAAGTTTGGGGAATATTACCAAACATCACAACAACAGCTAGCAGATCGGCCAGCCAAAATCCTGAGT
GTTACATGA

5- *actA*

>AB661448.1:7918-10176 Acinetobacter haemolyticus DNA, acinetoferrin biosynthesis gene cluster and transport genes, strain: ATCC 17906
ATGAAAGTTCATACACTTTCGACAACACTAACACTTCTAAGCTTGGCAGTAAGTGCCCAGCTTTATGCTC
AAACAACTGAGATCGATACCACGATTCAAAATAATGTTGCAGCAGAAGTCCAAAAACCAACACAACTTGC
GCCAATTGTTATGACAGCAACTCGTTCAGCACAAAGTATCGCTGAAATTGCAGGGACAGTTCAATCAATC
GAACAGAAACAAATTGGACAACAAGCGGCGGCTGGACGAAAGTTGGCAGATATTCTAGCTCAACTGGTTC
CATCACTCAGCCCGAGTAGCGGAACAACCACTAATTACGGTCAAACTATGCGTGGCCGTCAGGTGCTTGT
CCTTATTGATGGGGTTGCCCAAACAGGTTCACGTGATGCCGCACGCCAACTAAATAGTATTAGCCCTGAT
TCAATTGAAAAAATTGAGGTGGTTTCAGGTGCAAGTAGTATCTACGGTTCCGGTGCAACAGGCGGTATTA
TTAATATCATCACAAAGAAAGGGACTGGTGATGGTGTAAATTTTGAATCAAAACTTGGTGTTACATCTGG
CGATAATTTTAAAAATGATGCACTAGCTTATGAAGCTTATCAATCAGTTGGGTTTAATCAAGGTGATTGG
AGTGGTTTCTTAGGGGCAGGCTATACCAAACGTGGTGAAATCCAAGACAGTCATGGTAATCGCATTGGAC
CAGAGATTGCACAAACGGATCGCCAAGATACAGAAACGGTTGATGTGAATGGTCGTTTAAGCTGGCAATT
TACTGATACCCAAAAAATAAGTTTGGGGGCTCAGTATTATAATGACAAACAAGATTCAGAGTATGGTCCA
GATTATGGTCCAGACTTCGCAGTTTTAAAAGGCAATCCACCGAGTTTAAAAGCACTCAAAGGTTTTGAAA
TCGATGATCAGCCATTCACCAAACGTTATGATGTAAATACGCAATATCAAAACACGGATTTACTTGGGCA
AGAGCTGAATATAGAAGCATATTATCGCAATGAAAAAGCGCGCTTTTACCCGACGGTTTTAGCTAACTTT
ATTCCTGCTGGATATTATTTGGCTTACCAATCTGAGTCTGATATTGATGTTGCCGGTATTCGTGCTGCAA
TGACATCAAAGTTGAATGTTGCTGATCGTGATCTAAAACTGACTTATGGTATTGATTATGACCGTGAGAA
AGATAAGCAAACAGCTGATTTATATAGTTTTACCCACAATGGTCTGAAATATCAAAATACAGGGCGGAGT
TATGACTTTGGCCCAGATGCAACCATTAAAAACTTAGGTGCATTTGTTCAAGGAAATTATGATTTAACGG
ATGCATTGAATGTTCAAGCGGGCATTCGTTATCAACGTATTGAAAGCGACACCAGTGCATTTCAACCCAC
TGTTGCAGCTATTCAAGGTGATATCACCGGACAGCCGGTTGGAATGGTCGCTGCAGGTTCTGTTAAACAC
GACAAAACATTATTTAATTTAGGCGCTGTGTACAAATTAAATGACCAGCAACAAGTATTTGCAAACTTCT
CTCAAGGTTTTAGCTTACCAGATATTCAACGTGTATTACGCGATGTTTCTGCTGGTTATGTAGTTCGATC
AGGCAATGTTGATCCAATTACTGTCAATAGTTACGAATTGGGTTGGAGATTGCAAAATGACCTTGGTGCA
AATCTTGGATTAACAACGTTCTACAATACTTCTGATAAAGTCATCCAGTTTAAAGCTGATCGTTCTGTAA
CTGTTGCAGATACAGACCAACGTATTTATGGTCTTGAAGCGAATGCAAGCATGCCAGTATTGGATCAATT
TAGTGTCGGTGGGACATTATCTTATACTCGAGGTCAATTTAAAGACGCGAGTGGTTCTTGGCGAGAGCTG
AACGCTTTTCAAATTTCCCCAATCAAAGGAACATTATTCGGTGAGTGGAATGATGGCAAGGGCAATAGTC
TAAGAGTTCAGATGCTTGCCGTGAAAGGTACTGATAAAGCATATGAAGATTCCCTCGTTGCCAAATATGA
TGAAAATGTTCGCCCAAATGCGGCAACTAAAATCAAGGGGTATGCAGTCATGGATGTGATTGCCAATGCC
AAAGCAGGTCCTGGAACTGTAGGTTTTGGTGTCTATAATGTTTGGAATGCAGACTATAAAACGGTATATG
GCCAAGCGGCTGAAGCAGTTTATGGTCCAATTTCTAGTCTACCAGCTCAAGGTCGTACCTACGGTCTAAG
CTATACACTTAAATACTAA

6- *actB*

>AB661448.1:6305-6676 Acinetobacter haemolyticus DNA, acinetoferrin biosynthesis gene cluster and transport genes, strain: ATCC 17906
ATGAGCTTATATATCAATGAAATGCATATCCTAGACATGCGCTTTGCAGCGCATGTCTCGGTAGCAGAAT
TTGAACAATGGTTAGCACGAATTCAGCACTATTTCGAACATCAGCGAAATTTTGTGTTGATCATGCAAAC
TGATGAAAATACAGAATTTCCGGAGGAATATAGAGCAATCCAAGGCAAGTGGTATAAACAATATAAACAA
GATTTTTACCAATATTGCTTAGGTTTTGCGCGTATTGCTCAGGATGACGAAGATCGTATTCGATTGGATA
CACCAGCACTACACAAAGCTTGGCACGTCCCATATTTTGTCAGCGTAGATAAAACTGAAGCCATGCAATG

GGCGATGCAGAGGTATTTATGA

7- actC

>AB661448.1:6673-7908 Acinetobacter haemolyticus DNA, acinetoferrin biosynthesis gene cluster and transport genes, strain: ATCC 17906
ATGAATCAAAATTCATTAAATATTCAGAAAGTTTCAGCCTTAAGCCTAAGTGTCGTGGTGGTTTTATACC
TCGCGCATGCTTTACCACTGTATTTTTACAACGTGGCATTACCAGCGATTCTGCGCCATCAAGGTGTAGA
TTTACGTTGGATTGGCATGTTGTCGTTGCTTTATATTCCTTGGGCATTCAAATTCTTTTGGGCGCCTTTA
ATTGATCGTTTCTACTTAAAAAAACTAGGAAAGCGTAAGACTTGGTTGCTATTTACACAAATTGCACTGG
TGCTAGGTGTTGTTGCTTTGGCATTAACACAGTTTGATTATGGCCTCGGTGTATTTGTCATTGTTGGTTT
ATGGATTTCAACTTTTGCAGCAACTCAAGATATTGCCATTGATGGCTATACCGTCGAAACCTTTTCAGAG
TCTGAATATCGCTTAGGAAGCATGGCACAAAGCATCGGCGTTGCGCTCGGTAGTATGGTGGGTGGAGCAG
CAACTTTATGGCTTTACCAGCTTTATGGCTGGCAAACTGCATTGATTTCCTTAGCCGCAATGACGGCACT
GACTATGTTTGCGATTTTCCAAATCAAAGAAAAATCAATTTCTGAGAAAATCGCTAAACATCCACCAAGT
CTGATTCGTGCGTTTAAACGTCCAGAAATGTTATGGGCTTTAGCTTTGATCGTGTGTTATCGCTTCGTTG
AAGCACCTGCAATGGCAATGCTGAATCCGATGCTAATTGATCAAAAGTGGTCTTTGGCAGAGATTGGAGT
GTTGATGTCTGTGATCGGTGCGGGTATTGGATTATTAGCTGCTGTATCTGCTGCATTTCTCTTGAAAAAA
ATAGCAGCTACACAATTACTGATCTGGGCAGGCTGGGCACGTACTTTGATTTATGCCTTGTTGGGTGCAG
CTGTTCTACTCAGTTGGTTTGATCAATGGCACAGATTACTTGGTGTATTTGTAGTTGTCATTCTTGCAAT
TCGTTATATCGCGATGACGGCGTTATATGCACATTTCATGCAGACCAGCTCTAAAGATCAAGCAGGAACG
GATTTTACGATTTTGGTCTGTTTTGAATTATTGGTTTATTTCATTGGTGGTGCGATGTCAGGTTTCTTAG
CTAAAGCGTTTGGCTATGGAAATTTTTATTTAATTCTCGCCGCTGCATCCATTTTAAGCGTTTTACTCAG
TCAAGTTTTGATTTCTAAAGCAAAACAATCGAAACAACTTATCTAG

8- actD

>AB661448.1:10231-11457 Acinetobacter haemolyticus DNA, acinetoferrin biosynthesis gene cluster and transport genes, strain: ATCC 17906
ATGCAACAAAATTTTAAATTATTGGCTTGGGCAACTCTAGCCAACGGTACTTGTTTTTCAATGATTTTAC
CTTTACTTGCCCCATTAATTCGTGAGCTTGGCTTAACAGAAGTACAGGGCGGAGTAATTGTTTCAGCGGG
TGCAATTTGTATGGCGATTGCATCTATTCTGATTGCCCGAGGGGAAAAAATACAGACTCCTTATCAATTA
ATGAATTATGGTTTCTGGGGCATGACCATTACTTGGGCTATATTTACAGCGATTCTTTATTGGGGAATAC
AACAAACATTACCCGTAATGATAGTTTTTGCTTTGTTGGTGATTAGTCGAGCGAGTACTGGTGGTTTTAT
GGCTATGCCACAAATTGGTCTACAAAGTTATGTCATGCAACATGTCACAGAGGAACAACAACGCAGTCAA
AAAATGGCAATGTATGGTGCGATGAATAGCTTAGGTATGATCGTCGGTCCATTTCTGACGTCCGTGTTAT
TGTTTGGTGGCATTCTATTGCCAATGTGGATTGCTGTTATTTTGTTAGTGATTTTTAGTTTGATTATCAC
AATGTATTTTAAACATGATGAGGGTAAACAAAACACAATATTGGATCAAAAACAGCCTGTAAAAATAGAG
CATTCATTTTCATTAAAACCAGCTATGCCTTGGCTTATTTTAGGATTTACTACCTATATGGCTATCGTGA
CGCTCAATATGACTGCAGGATTCTATATTCAAGACCATTTTCATTTAAGCTCACAACAAAGTGCGATTTA
TTTTTCCCAATGTATGCTCATTGTTGGTTTTAGCTTAGTGATTACTCAGATCCTGATTGTAAAAGTACTT
CATTTAAAACTAAAAGCTTTGGTATTGCTGGGTACGTTTAGCATGGTTAGTGGACTTATCCTATCTTTAA
GTGCAATCCATATTTGGATGTTTCAAATAAGTTATTTATTTTATGGGGTCGGGGTGGCAAGCCTTTTGCC
TGCATTTACCACAGGGGCGGCTCAATCTGTTCCGCAAACTGCTCAAGTCAAAATGGCAAGTATTTGTACA
GCTACTCAAGCGATTGGTCTCATTGTGGCGCCTTTACTGAGCACTTTTCTATATCAGTTCGGCATTCAAT
TACCTTTTTATTTACTCTTAATATTGATGATTGCTGTGGCGATCTACTTGTTGATCATATCCTATTCAAA
TCAGAATACGATGATAAGCAATAAGGTTAACTCCTAA

Protein sequence

>WP_113997333.1 acinetoferrin biosynthesis protein AcbA [Acinetobacter haemolyticus]
MQSLANRLALQHFVNAYTQETGKGHLLHKSQQSLQLQTFSQGLTLLSIPITSIQARCFFPLSYVSCVGRH
RLADLPQIIIDEKIKVFSPVAIVGLLLEELVQEAPVHLDAASLVEKWIQSRDALQQFLQNREYEFDDLVK
AGQSFIETEQALILGHSMHPAPKSRTGFVHEDWLNFSPENKGKTQLHYWLVHQDYIAEGSATDEPISAQL
KTALQWYLSENDLNLLKTHAEYKLLPLHPWQARYLQGKAWFERLKQTGQLIDLGLRGWQFSPTTSIRTLA
SFNAPWMVKPSLSVMITNSIRVNLAKECHRGELTHRLWHSEFGQNILKQCPSLKAVNDPAWIALKIDDEV
INDSICIFRDQPFHPQQQVTCIASLCQDHPIKPLNRFNALFAKIAKAHPDAEVSEIASDWFNRFLEVSLQ
PLMYLYHRYGMAFESHQQNVLLELENHFPKTLWLRDNQGFYYIEEFATEILQALPELKEKAFAVGPKDFV
DERFSYYFFGNTLFGIINAIGATGYITEDELLAHLTGFLEEQLQQYPESTLIQGLLFNSTLPYKGNLLTR
LHELDELIAPVENQSVYVQLPNPLRISQKDVSYA

>WP_456691042.1 acinetoferrin biosynthesis monooxygenase AcbB [Acinetobacter haemolyticus]
MLDFIGIGLGPFNLSLASLLQNKSSLNYVFFEQKAQFDWHAGMQLPNTVLQVPFMADLVSMVDPTSPFSF
LNYLRHQQRLYKFYFLEQPHIPRCEYNHYCQWVAEQLDCIEYQSQVLKIEPQTIGFKVVVESNGVQQSYL
CRHLVIGSGNVPYLPECFAKIQKVLPQQCLHSAHYMTHAATDLHGDVVVLGSGQSAAEVFIDLFDEQQDT
DNHQFDLHWFTRSQGFFPMEYAPLGLEHFSPDYAQHFYDLSAQQKEQQLKQQALLYKGISAKTIREIYQR
LYHRSIAGQSIQTHLHSQCDLKDAEVLDTQKIRLHFEHRATAQSFHLDCDFLVAATGYFTPDFAFMQLLK
PYIEFDHKQRWQITEDYRVVHRLNGHIFVQNQEMHSHGVGTPDLGLGAYRAATIINQLLAEPLYELGNQA
QTFQHFNLSQNPKICMVDDKAQSKECVCENSANKNHSIFKKTEQNVMTTHPNSRGANTRKQTASVHSTYE
KLI

>WP_436872332.1 acinetoferrin biosynthesis protein AcbC [Acinetobacter haemolyticus]
MNFATLKINQQQWRAAGQRLIEMAIAEFLYEEIIEVKALSAGRYRLDLGNRQYEFQGHQYLLGHWNIQEA
SVRDVTHSQENDQPAWNLHEFIVAMSDSSNVKPFTKAYLIKEMNNTWLAEAHLFNETRLPSIAVLTEPHY
KVEGMLRGHPWLIMSKGRMGFGYDDYLSAAPELSPEVKVLWLAVHRDLAEYRSTEDWSACGLYQHEFDAN
ELQQFINILQEKNLDPQNYFLIPVHAWQWHQWLVPTYANEIVDQKIIELNISQDSYVPMQSIRTLCNTSN
LQRHYIKLPVSIFNTAVYRGLPSKRNLAAPAVTAWLKQIHQQDQDLQNTGVIFLGEVATLTIHQPCFDRI
DGAPYQFKELFGCLWRESVDRYVDSSQQVLSQAALLHRDISGQSILSVLIQASGLSPLAWLAQFAQVSMS
PLLLCLYRYGLAFSPHGENTMLVHENGVPKAMVLKDFIDDINLVDEDFPELIQLPSEADLLLRHEATDLS
HFIFTGLFMVHYRYICNVFLQDYPEYSELDFWQTISNTIVEFNQKHPELAERADKFAMLRPSYTKICLNR
VRLFTTSYNDEAERPVPVFLDPIANPVSPETLKTWAQQPRQAKVG

>WP_429643868.1 acinetoferrin biosynthesis acetyltransferase AcbD [Acinetobacter haemolyticus]
MKTISQQLPDYFEYFEDGTQYYLRQVQYPQDIPLLHKWMHEPHVIPQWQLNKSELELQVYFDKMLADDHH
RLLIVGIDGKDVGYTEIYEGKRDRLGRYYDGDDNDLGWHLLFGDKSVFGKGFLRPTIRLLSFYIFEHSKA
KKIVGEPDHTVKPYAAVVAELCYESQRLIPMPEKTAMLYYCFRETFYNKFGDYYQTSQQQLANQPAKLLS
VT

>WP_347458811.1 putative TonB-dependent ferric acinetoferrin receptor ActA [Acinetobacter haemolyticus]
MKVHTLSTTLTLLSLAVSAQLYAQTTEIDTTIQNNVAAEVQKPTQLAPIVMTATRSAQSIAEIAGTVQSI
EQKQIGQQAAAGRKLADILAQLVPSLSPSSGTTTNYGQTMRGRQVLVLIDGVAQTGSRDAARQLNSISPD
SIEKIEVVSGASSIYGSGATGGIINIITKKGTGDGVNFESKLGVTSGDNFKNDALAYEAYQSVGFNQGDW
SGFLGAGYTKRGEIQDSHGNRIGPEIAQTDRQDTETVDVNGRLSWQFTDTQKISLGAQYYNDKQDSEYGP
DYGPDFAVLKGNPPSLKALKGFEIDDQPFTKRYAVNTQYQNTDLLGQELNVEAYYRNEKARFYPTVLANF

IPAGYYLAYQSESDIDVAGIRAAMTSKLNVADRDLKLTYGIDYDREKDKQTADLYSFTHNGLKYQNTGRS
YDFGPDATIKNLGAFVQGNYDLTDALNVQAGIRYQRIESDTSAFQPTVAAIQGDITGQPVGMVAAGSVKH
DKTLFNLGAVYKLNDQQQVFANFSQGFSLPDIQRVLRDVYAGYVVRSGNVDPITVNSYELGWRLQNDLGA
NLGLTTFYNTSDKVIQFKADRSVTVADTDQRIYGLEANASMPVLDQFSVGGTLSYTRGQFKDASGSWREL
NAFQISPIKGTLFGEWNDDKGNSLRVQMLAVKGTDKAYEDSLVAKYDENVRPNAATKIKGYAVMDVIANA
KAGPGTVGFGVYNVWNADYKTVYSQAAEAVYGPISSLPAQGRTYGLSYTLKY

>WP_429643871.1 protein ActB [Acinetobacter haemolyticus]
MSLYINEMHILDMRFAAHVSVAEFEQWLARIQHYFEHQRNFVLIMQTDANTEFPEEYRAIQGKWYKQYKQ
DFYQYCLGFARIAQDEEDRIRLDTPALHKAWHVPYFVSVDKTEAMQWAMQRYL

>WP_436872333.1 putative ferric acinetoferrin MFS transporter permease subunit ActC [Acinetobacter haemolyticus]
MNQNSLNIQKVSALSLSVVVVLYLAHALPLYFYNVALPAILRHQGVDLRWIGMLSLLYIPWAFKFFWAPL
IDRFYFKKLGKRKTWLLFTQIALVLGVVALALTQFDYGLGVFVIVGLWISTFAATQDIAIDGYTVETFSE
SEYRLGSMAQSIGVALGSMVGGAATLWLYELYGWQTALISLAAMTALTMLAIFQIKEKSNVEKISKQPPS
LIRAFKRPEMLWALALIVCYRIVEAPAMAMLNPMLIDQKWSLAEIGVLMSVIGAGIGLLAAVSAAFLLKK
IAATQLLIWAGWARTLIYTLLGAAVLLSWFDQWHRLLGVFVVVILAIRYIAMTALYAHFMQTSSKDQAGT
DFTILVCFELLVYFIGGAMSGFLAKAFGYGNFYLILAAASILSVLLSQVLISKAKQSKQLI

>WP_456691043.1 putative acinetoferrin export transporter ActD [Acinetobacter haemolyticus]
MQQNFKLLAWATLANGTCFSMILPLLAPLIRELGLTEVQGGVIVSAGAICMAIASILIARGEKIQTPYQL
MNYGFWGMTITWAIFTAILYWGIQQTLPVMIVFALLVISRASTGGFMAMPQIGLQSYVMQHVTEEQQRSQ
KMAMYGAMNSLGMIVGPFLTSVLLFGGILLPMWIAVILLVIFSLIITMYFKHDEGKQSTILDQKQPVNVE
HSFSLKPAMPWLILGFTTYMAIVTLNMTAGFYIQDHFHLSSQQSAIYFSQCMLIVGFSLVITQILIVKVL
HLKLKALVLVGTFSMVSGLILSLSAIHIWMFQISYLFYGVGVASLLPAFTTGAAQSVPQTAQVKMASICT
ATQAIGLIVAPLLSTFLYQFGIKLPFYLLLILMIAVAIYLLIISYSNQNTMISNKVNS

II- Genes encode Desferrioxamine E pathway

1- desA

>AL939114.1:1191-2633 Streptomyces coelicolor A3(2) complete genome; segment 11/29
ATGCGCTCGCACCTGCTCAACGACACCACCGCGGAGCAGTACCGCCGTTCCGTGACCGAAGGAGTCGAGC
GGGTGGCCGCCAAACTCGCCACCACCGACCGCCCGTTCACCGGCGTCACGGTCGACGCCCTCTCCCCCCG
CATCGACGCGATCGACCTCGACGAGCCGCTGCACGACACCGCCGCGGTCCTCGACGAGCTGGAGGACGTC
TACCTCCGCGACGCCGTCTACTTCCACCACCCCCGCTACCTCGCCCACCTCAACTGCCCGGTCGTCATAC
CGGCGCTGCTCGGTGAAGCGGTCCTGTCCGCCGTCAACTCCTCCCTGGACACCTGGGACCAGTCGGCCGG
CGGCACGCTCATCGAGCGGAAGCTGATCGACTGGACCTGCGCCCGCATCGGCCTCGGCCCGGCGGCCGAC
GGCGTGTTCACCTCCGGCGGCACCCAGTCCAACCTCCAGGCGCTGCTCCTCGCCCGCGAGGAGGCGAAGG
CCGAGGACTTCGCCGACCTGCGGATCTTCGCCTCCGAGGCCAGCCACTTCAGCGTCAGGAAGTCCGCGAA
ACTGCTCGGCCTCGGCCCCGACGCCGTCGTGTCGATCCCGGTCGACCGCGACAAGCGGATGCAGACCGTC
GCCCTCGCCCGCGAGCTGGAGCGCTGCGCGCGGGACGGCCTGGTCCCCATGGCCGTCGTCGCCACCGGCG
GCACCACCGACTTCGGCTCGATCGACCCGCTGCCGGAGATCGCCGGGCTGTGCGAGCAGTACGGCGTGTG
GATGCACGTCGACGCGGCCTACGGCTGCGGGCTGCTCGCCTCCCTGAAGTACCGGGACCGCATCACCGGC
ATCGAGCGGGCCGACTCGGTCACCGTGGACTACCACAAGTCCTTCTTCCAGCCGGTGAGTTCGTCGGCCG
TGCTGGTCCGGGACGCGGCCACCCTGCGCCACGCCACCTACCACGCGGAGTACCTCAACCCGCGCCGCAT
GGTGCAGGAACGTATCCCCAACCAGGTGGACAAGTCCCTCCAGACCACCCGCCGCTTCGACGCGCTCAAG
CTGTGGATGACGCTGCGCGTGATGGGCGCCGACGGCATCGGCGTCCTCTTCGACGAGGTGTGCGACCTGG
CCGCCGAGGGCTGGAAACTGCTCGCCGCCGACCCGCGCTTCGACGTCGTGGTCCAGCCGTCGCTGTCCAC
GCTGGTCTTCCGCCACATCCCGGCGGACGTCACCGATCCCGCCGAGATCGACCGCGCCAACCTGTACGCC
CGCAAGGCCCTGTTCGCGTCCGGCGACGCCGTGGTCGCGGGCACCAAGGTCGCCGGTCGCCACTACCTGA
AGTTCACCCTGCTCAACCCCGAGACGACCCCGGCCGACATCGCCGCCGTCCTCGACCTGATCGCCGGCCA
CGCCGAGCAGTACCTGGGAGACTCCCTTGACCGCGCTTCCTGA

2- desB

>AL939114.1:2656-3897 Streptomyces coelicolor A3(2) complete genome; segment 11/29
GTGGGCATCGGCCTCGGCCCCTTCAACCTCGGCCTCGCCTGCCTCACCGAGCCCGTCGCCGAGCTGAACG
GCGTCTTCCTGGAGTCCAAGCCGGACTTCGAGTGGCACGCCGGGATGTTCCTGGACGGCGCCCACCTCCA
GACCCCGTTCATGTCGGACCTGGTGACGCTCGCCGACCCGACCTCGCCGTACTCCTTCCTCAACTACCTG
AAGGAGCAGGGCCGGCTGTACTCCTTCTACATCCGGGAGAACTTCTACCCGCTGCGGGTCGAGTACGACG
ACTACTGCCGCTGGGCCGCGCGCAAGCTGAGCAGCGTCCGCTTCTCCACCACGGTCACCGAGGTCACGTA
CGACGAGCGCGAGGAGCTGTACGCCGTCGCCACCACGTCCGGCGACACCTACCGCGCCCGCCGCCTCGTC
CTCGGCACCGGCACCCCGCCGCACATCCCGGACGCCTGCCGGGGCCTGGCCGGCGACTTCCTCCACAACT
CCCGGTACGTGCGGCACCGGGCGGAGCTGGTGAAGAAGAAGTCGATCACGCTGGTCGGCAGCGGCCAGTC
CGCCGCCGAGATCTACCAGGACCTGCTGAGCGAGATCGACGTCCACGGCTACGGGCTGAACTGGGTGACC
CGCTCCCCGCGGTTCTTCCCGCTCGAATACACCAAGCTCACCCTGGAGATGACGTCCCCGGAGTACGTGG
ACTACTACCACGCGCTGCCCGAGGACACCCGCTACCGCCTCACGGCCGAGCAGAAGGGCCTGTTCAAGGG
CATCGACGGCGACCTGATCAACGAGATCTTCGACCTGCTCTACCAGAAACGGCTCGGCGGCCCCGTCCCC
ACCCGCCTGCTCACCAACTCGGCGCTGACCAGCGCCCGGTATGCGGACGGCACCTACACGCTCGGGTTCC
GCCAGGAGGAGCAGGGCACGGACTTCGAGATCGAGACCGAGGGCCTGGTCCTCGCCACCGGCTACCGGTA
CACCGAGCCGGAGTTCCTCAAGCCCGTCCGGGACCGGCTGCGCTACGACTCCCGCGGCAACTTCGACATC
GGCCGCAACTACGCCGTCGACGTCACGGGAGGCGGCGTGTTCCTCCAGAACGCGGGGGTCCACGCGCACA
GCGTCACCAGCCCCGACCTGGGCATGGGCGCCTACCGCAACAGCTGCATCGTCCGGGAGCTGCTCGGACG
CGAGTACTACCCGGTCGAGCAGTCGATCGCGTTCCAGGAGTTCGCCGTATGA

3- desC

>AL939114.1:3894-4448 Streptomyces coelicolor A3(2) complete genome; segment 11/29
ATGAGCCGCTTGAGCACCACCACCCCCGTCGGGGCACTGACCCTGCGCCCCGTCGACCCGCTGACGGACG

CCGTACTGCTGCACGGCTGGCTCACCCACCCCAAGTCCGCGTTCTGGATGATGCAGGACGCCCGGCTGGT
GGACGTCGAGCGGGCCTACATGGAGCTGGCCGCCGACGAGCACCAGCAGGCCCACCTCGGCCTGCACGAC
GGGGTCCCGGCCTTCCTGACGGAGCGCTACGACCCCGCCCACCGCGAACTGGTCGGGCTGTACGAGCCCG
AGCCGGGCGACGTCGGCATGCACTTCCTGGTCGCGCCCACCGACCGGCCCGTGCACGGCTTCACCCGCGC
CGTGATCACCACCGTGATGACGGAGCTGTTCGCCGACCCGGCGACCCGGCGGTCGTCGTCGAACCGGAC
GTCACCAACACCGCCGTGCACGCCCTGAACGCAGCCGTCGGATTCGTGCCCGAGCGCGAGATCCAGAAGC
CGGAGAAGAAGGCCTTGCTGAGCTTCTGCACCCGCGAGCAGTTCGCGAAGGCGGTGTCCGCATGA

4- desD

>AL939114.1:4445-6232 Streptomyces coelicolor A3(2) complete genome; segment 11/29

ATGAGCCTCGCCGACGCCGTCGCCCACCTGACCCCCGAACGCTGGGAGGAGGCCAACCGCCTCCTGGTCC
GCAAGGCGCTGGCCGAGTTCACCCACGAGCGGCTGCTGACCCCCGAGCGGGAGCCGGACGACGGGGGCGG
GCAGACGTACGTCGTCCGCAGCGACGACGGCCAGACCGCGTACCGCTTCACCGCCACCGTCCGCGCCCTG
GACCACTGGCAGGTGGACGCCGCCTCCGTCACCCGCCACCGCGACGGCGCGGAACTCCCGCTCGCCGCGC
TGGACTTCTTCATCGAGCTGAAGCAGACCCTGGGCCTGAGCGACGAGATCCTCCCGGTCTACCTGGAGGA
GATCTCCTCCACCCTCTCCGGCACCTGCTACAAACTGACCAAGCCGCAGCTCAGCTCCGCCGAGCTGGCC
CGGAGCGGAGACTTCCAGGCCGTCGAGACGGGCATGACCGAGGGCCACCCGTGCTTCGTCGCCAACAACG
GGCGCCTCGGCTTCGGCATCCACGAGTACCTGTCGTACGCCCCCGAGACCGCGAGCCCGGTCCGGCTGGT
GTGGCTGGCCGCGCACCGCTCGCGGGCGGCGTTCACGGCGGGCGTGGGCATCGAGTACGAGTCCTTCGTC
CGGGACGAGCTGGGCGCGGCCACCGTCGACCGCTTCCACGGCGTGCTGCGCGGGCGCGGCCTGGACCCGG
CCGACTACCTGCTCATCCCGGTCCACCCCTGGCAGTGGTGGAACAAGCTCACCGTCACCTTCGCCGCCGA
GGTCGCCCGCGGGCACCTGGTGTGCCTGGGCGAGGGCGACGACGAGTACCTGGCCCAGCAGTCCATCCGC
ACCTTCTTCAACGCCTCGCACCCCGGGAAGCACTACGTGAAGACTGCCCTGTCCGTCCTCAACATGGGCT
TCATGCGCGGTCTGTCGGCGGCGTACATGGAGGCCACTCCGGCCATCAACGACTGGCTCGCCCGGCTGAT
CGAGGGCGACCCGGTGCTGAAGGAGACGGGGCTGAGCATCATCCGGGAGCGGGCGGCCGTCGGCTACCGG
CACCTGGAGTACGAGCAGGCCACCGACCGCTACTCGCCCTACCGCAAGATGCTGGCGGCGCTGTGGCGGG
AGAGCCCGGTGCCGTCCATCCGGGAGGGCGAGACGCTCGCCACCATGGCCTCCCTGGTCCACCAGGACCA
CGAGGGCGCCTCCTTCGCGGGCGCGCTGATCGAGCGGTCCGGACTCACGCCCACCGAGTGGCTGCGGCAC
TACCTGCGGGCCTACTACGTCCCGCTGCTGCACAGCTTCTACGCCTACGACCTGGTGTACATGCCGCACG
GCGAGAACGTGATCCTGGTGCTGGCGGACGGGGTGGTGCGGCGGGCGGTCTACAAGGACATCGCCGAGGA
GATCGCGGTGATGGACCCGGACGCGGTGCTGCCGCCGGAGGTCTCCCGCATCGCGGTGGACGTGCCGGAC
GACAAGAAGCTCCTGTCGATCTTCACGGACGTCTTCGACTGCTTCTTCCGCTTCCTGGCCGCGAACCTGG
CGGAGGAGGGGATCGTCACGGAGGACGCCTTCTGGCGGACGGTCGCGGAGGTCACCCGGGAGTACCAGGA
GTCGGTGCCGGAGCTGGCCGACAAGTTCGAGCGGTACGACATGTTCGCGCCCGAGTTCGCCCTGTCCTGC
CTCAACCGGCTCCAGCTGCGCGACAACCGGCAGATGGTGGACCTGGCCGACCCGTCCGGCGCGCTCCAGC
TCGTCGGCACCCTGAAGAACCCCCTGGCAGGCCGGTAG

4- desE

>CP181172.1:3033412-3034461 Streptomyces coelicolor strain M1152 chromosome, complete genome

ATGTCCCACGCCAGCGCTACCCACCCGACCCGCCGTGGAATCCTCGCCGCCGGCGGCGCCCTCGGCCTCG
GCGCCGTGCTCGCGGCCTGCGGCGACGGCGACGGCAAGAGCGATGGGGCGGGCGACGGGTCGGGCGGCGC
CGCCAAGTCCGGCCCCTGGTCCTTCAAGGACGACCGCGGCACGACCGTGAAGCTGGACAAGGTGCCGGCG
AACATCGTCGCCTTCACGGGTGTCGCCGCCGCCCTCTTCGACTACGGCGTCGAGGTCAAGGGCGTCTTCG
GCCCGACCACCACCAAGGACGGCAAGCCCGACGTGCAGGCCGGCGACCTCGACGTCGACAAGGTCACCGT
GCTCGGCAACGAGTGGGGCAAGCTCAACGTCGAGAAGTACGCCTCCCTCGCCCCGGAGGTGCTCATCACC
ACGACGTTCGACACCGCGGGCACCCTGTGGTCCGTCCCGGAGGAGTCGAAGGACAAGGTCGCCAAGCTCG
CCCCGAGCGTCGCGATCTCGGTCTTCGACCGCCAGCTCACCCAGCCGCTCCAGCGCATGTGGGAGCTGGC
GGAGTCGCTCGGCGCGGACATGAAGGCCAAGAAGGTCACCGACGCCAAGGCCGCCTTCGACAAGGCCGCC
GCCCGGCTGCGCGCCGCCGCCAAGGCCAAGCCGGAGATCCGGGTGCTGGCCGGTTCCGCGAGCCCCGACC
TGTTCTACGTCTCCGGCACCAACCTCTCGGTGGACCTGGAGTACTTCAAGGCCCTCGGCGTGAACTTCGT
CGAGCCCTCCGAGGACGCCAAGAAGGCGACCGGCGGCTGGTTCGAGTCCCTGAGCTGGGAGAACGTCGAC
AAGTACCCGGCCGACGTCATCATCATGGACGACCGCGCCTCGACCATCCAGCCCGCCGACATCACCGAGG
GCACCTGGAAGCAGCTCCCCGCGGTCAAGGCCGGACAGGTCATCGCCCGCTCCCCCGAGCCGATCCTGTC

CTACGACAAGTGCACGCCGCTCCTCGACAACCTGGCCGAGGCGATCGAGAACGCCAAGAAGGTCGGCTGA

5- desF

>AL939114.1:182-1027 Streptomyces coelicolor A3(2) complete genome; segment 11/29
ATGCGCGTGCCAGACCCCGCGCCCCCGGCAGGATCCAAACGACACCCCCCAAGCCCGGCCCTGGCCCGGG
TCACCTTCGCGGGGCCCGACCTGCGCGCCTTCCGCTCCGACGGCCTCGACCAGTCGCTGTCGCTGTTCCT
GCCGCACCCGGGGCAGGCGGAGCCCGCGGTCCCGGTGGAGCTGGGCGAGGGCTGGTGGCAGGGCTGGCGG
GAACTGCCGGAGGACGTACGGGCGGTGATGCGCTCGTACACGCTGCGGTCACTGCGCCGCGACACCGACG
GGCACACCGCCGAGATCGACGTCGACTTCGTCCTGCACGGCCTCGAACCGGACTCGGGGATCCAGGCCGG
TCCCGCCGCCCGCTGGGCCGCCGACGCCGCCCCCGGCGACCGCGTGCTGCTGCTCGGCCCGGCGGTCGCC
GACAACCGGGCGATCCGCTTCCGGCCGCCCGAGGACACCGACCTGGTGGTGATCTGGGGTGACGAGACAG
CCGTACCCGCCGCCTGCGCCATCGTGGAGGCACTGCCGGCCGGCACCCGCGCGCGGGTCTGGCTTCAGGT
GCCGCACGCCGAGGACGTGCAGGACCTCCGGACGGCCGCGGACGCCGAGATCACCTGGCTGGTCGGGGAC
GCCGCCGACGGGCCCGAGGCGACCCTCGCCACCCTCCGCGCCGCCCAACTGCCGCCCGCCGAACACCCCT
ACGTCTGGATCGCGGGCGAGTCCGGCTGCGTGAAGCGGCTGCGGCGGCACTTCGTGGGCGAGCGCGGCGT
CGACCGACGGCGCGTCACCTTCGTCGGCTACTGGCGCCGGGGCCTGACGGAGGAACAACTCCGCGAGCAG
GGCTGA

Protein sequence

>WMT33446.1 lysine decarboxylase DesA [Streptomyces coelicolor]
MRSHLLNDTTAEQYRRSVTEGVERVAAKLATTDRPFTGVTVDALSPRIDAIDLDEPLHDTAAVLDELEDV
YLRDAVYFHHPRYLAHLNCPVVIPALLGEAVLSAVNSSLDTWDQSAGGTLIERKLIDWTCARIGLGPAAD
GVFTSGGTQSNLQALLLAREEAKAEDFADLRIFASEASHFSVRKSAKLLGLGPDAVVSIPVDRDKRMQTV
ALARELERCARDGLVPMAVVATGGTTDFGSIDPLPEIAGLCEQYGVWMHVDAAYGCGLLASLKYRDRITG
IERADSVTVDYHKSFFQPVSSSAVLVRDAATLRHATYHAEYLNPRRMVQERIPNQVDKSLQTTRRFDALK
LWMTLRVMGADGIGVLFDEVCDLAAEGWKLLAADPRFDVVVQPSLSTLVFRHIPADVTDPAEIDRANLYA
RKALFASGDAVVAGTKVAGRHYLKFTLLNPETTPADIAAVLDLIAGHAEQYLGDSLDRAS

>CAB87218.1 hypothetical protein [Streptomyces coelicolor A3(2)](DesF)
MRVPDPAPPAGSKRHPPSPALARVTFAGPDLRAFRSDGLDQSLSLFLPHPGQAEPAVPVELGEGWWQGWR
ELPEDVRAVMRSYTLRSLRRDTDGHTAEIDVDFVLHGLEPDSGIQAGPAARWAADAAPGDRVLLLGPAVA
DNRAIRFRPPEDTDLVVIWGDETAVPAACAIVEALPAGTRARVWLQVPHAEDVQDLRTAADAEITWLVGD
AADGPEATLATLRAAQLPPAEHPYVWIAGESGCVKRLRRHFVGERGVDRRRVTFVGYWRRGLTEEQLREQ
G

>CAB87220.1 putative monooxygenase [Streptomyces coelicolor A3(2)](DesB)
MGIGLGPFNLGLACLTEPVAELNGVFLESKPDFEWHAGMFLDGAHLQTPFMSDLVTLADPTSPYSFLNYL
KEQGRLYSFYIRENFYPLRVEYDDYCRWAARKLSSVRFSTTVTEVTYDEREELYAVATTSGDTYRARRLV
LGTGTPPHIPDACRGLAGDFLHNSRYVRHRAELVKKKSITLVGSGQSAAEIYQDLLSEIDVHGYGLNWVT
RSPRFFPLEYTKLTLEMTSPEYVDYYHALPEDTRYRLTAEQKGLFKGIDGDLINEIFDLLYQKRLGGPVP
TRLLTNSALTSARYADGTYTLGFRQEEQGTDFEIETEGLVLATGYRYTEPEFLKPVRDRLRYDSRGNFDI
GRNYAVDVTGGGVFLQNAGVHAHSVTSPDLGMGAYRNSCIVRELLGREYYPVEQSIAFQEFAV

>CAB87221.1 putative aceytltranferase [Streptomyces coelicolor A3(2)](DesC)
MSRLSTTTPVGALTLRPVDPLTDAVLLHGWLTHPKSAFWMMQDARLVDVERAYMELAADEHQQAHLGLHD
GVPAFLTERYDPAHRELVGLYEPEPGDVGMHFLVAPTDRPVHGFTRAVITTVMTELFADPATRRVVVEPD
VTNTAVHALNAAVGFVPEREIQKPEKKALLSFCTREQFAKAVSA

>WMT33449.1 desferrioxamine E synthetase DesD [Streptomyces coelicolor]
MSLADAVAHLTPERWEEANRLLVRKALAEFTHERLLTPEREPDDGGGQTYVVRSDDGQTAYRFTATVRAL
DHWQVDAASVTRHRDGAELPLAALDFFIELKQTLGLSDEILPVYLEEISSTLSGTCYKLTKPQLSSAELA
RSGDFQAVETGMTEGHPCFVANNGRLGFGIHEYLSYAPETASPVRLVWLAAHRSRAAFTAGVGIEYESFV
RDELGAATVDRFHGVLRGRGLDPADYLLIPVHPWQWWNKLTVTFAAEVARGHLVCLGEGDDEYLAQQSIR
TFFNASHPGKHYVKTALSVLNMGFMRGLSAAYMEATPAINDWLARLIEGDPVLKETGLSIIRERAAVGYR
HLEYEQATDRYSPYRKMLAALWRESPVPSIREGETLATMASLVHQDHEGASFAGALIERSGLTPTEWLRH
YLRAYYVPLLHSFYAYDLVYMPHGENVILVLADGVVRRAVYKDIAEEIAVMDPDAVLPPEVSRIAVDVPD
DKKLLSIFTDVFDCFFRFLAANLAEEGIVTEDAFWRTVAEVTREYQESVPELADKFERYDMFAPEFALSC
LNRLQLRDNRQMVDLADPSGALQLVGTLKNPLAGR
